# Abstract conceptual feature ratings: the role of emotion, magnitude, and other cognitive domains in the organization of abstract conceptual knowledge

**DOI:** 10.3389/fnhum.2013.00186

**Published:** 2013-05-23

**Authors:** Sebastian J. Crutch, Joshua Troche, Jamie Reilly, Gerard R. Ridgway

**Affiliations:** ^1^Dementia Research Centre, Department of Neurodegenerative Disease, UCL Institute of Neurology, University College LondonLondon, UK; ^2^Stroke Unit, Northwick Park HospitalHarrow, London, UK; ^3^Department of Speech, Language and Hearing Sciences, University of FloridaGainesville, FL, USA; ^4^Eleanor M. Saffran Center for Cognitive Neuroscience, Temple UniversityPhiladelphia, PA, USA; ^5^Wellcome Trust Centre for Neuroimaging, UCL Institute of Neurology, University College LondonLondon, UK

**Keywords:** abstract conceptual knowledge, emotion, quantity, multidimensional scaling

## Abstract

This study harnessed control ratings of the contribution of different types of information (sensation, action, emotion, thought, social interaction, morality, time, space, quantity, and polarity) to 400 individual abstract and concrete verbal concepts. These abstract conceptual feature (ACF) ratings were used to generate a high dimensional semantic space, from which Euclidean distance measurements between individual concepts were extracted as a metric of the semantic relatedness of those words. The validity of these distances as a marker of semantic relatedness was then tested by evaluating whether they could predict the comprehension performance of a patient with global aphasia on two verbal comprehension tasks. It was hypothesized that if the high-dimensional space generated from ACF control ratings approximates the organization of abstract conceptual space, then words separated by small distances should be more semantically related than words separated by greater distances, and should therefore be more difficult to distinguish for the comprehension-impaired patient, SKO. SKO was significantly worse at identifying targets presented within word pairs with low ACF distances. Response accuracy was not predicted by Latent Semantic Analysis (LSA) cosines, any of the individual feature ratings, or any of the background variables. It is argued that this novel rating procedure provides a window on the semantic attributes of individual abstract concepts, and that multiple cognitive systems may influence the acquisition and organization of abstract conceptual knowledge. More broadly, it is suggested that cognitive models of abstract conceptual knowledge must account for the representation not only of the relationships between abstract concepts but also of the attributes which constitute those individual concepts.

Much of the debate surrounding embodied and disembodied theories of cognition has concerned whether sensorimotor processing plays a fundamental, interactive or epiphenomenal role in conceptual knowledge (as outlined in more detail in other papers in this Research Topic). This debate has recently been framed or re-framed as an embodiment continuum or “graded grounding,” highlighting the similarities and differences between so-called strong and weak forms of the embodiment hypothesis (Chatterjee, 2010; Dove, 2011; Meteyard et al., 2012; see also Kiefer and Pulvermüller, 2012). One notable feature of some weak embodiment theories is their emphasis upon the contribution to abstract concepts of not only motor and sensory information but also emotion information [e.g., Andrews et al., 2009; Kousta et al., 2009, 2011; Newcombe et al., 2012; see Pecher et al. (2011), for a review]. Such authors acknowledge that not all abstract words are affectively loaded, but suggest that the acquisition of such affectively loaded concepts provides a framework for the subsequent acquisition of non-affective concepts based on linguistic experience alone (Meteyard et al., 2012).

Motivations for inclusion of emotion information include the fact that most emotion words refer to abstract states, and also that emotional development precedes language development (Bloom, 1998). However, many other cognitive systems also demonstrate development prior to language acquisition. Although emotion does appear to represent a core primitive that is evident prior to proficient language use, the same can be said for many other cognitive skills (e.g., novelty detection). Thus, the focus on emotion as a latent factor driving abstract word representation may in fact present only a portion of the variance of the complex phenomenon.

We have recently reported a new approach to examining abstract conceptual attributes, in which multidimensional ratings are used to evaluate the contribution not only of sensory, motor and emotion information but also of a range of additional types of information (Crutch et al., 2012). Just as motor information represented by activity in the motor, premotor, and supplementary motor areas is hypothesized to be particularly important in the formation and activation of certain concepts (e.g., actions, tools; Hauk et al., 2004; Garcea and Mahon, 2012), so it is hypothesized that other cognitive domains might contribute differentially to the acquisition and organization of abstract concepts. In other words, it is proposed that affect is not the only aspect of internal experience (other than linguistic experience) that contributes to the formation and organization of abstract conceptual knowledge. The additional types of information considered include social interaction, morality, executive function, quantity, time, space, and polarity.

The social interaction dimension was selected following previous work on the “words as tools” (WAT) proposal that social and linguistic information are particularly important in the acquisition of abstract terms (e.g., Borghi et al., 2011; Scorolli et al., 2011), and evidence suggesting the importance of introspection for the development of such concepts (e.g., Barsalou, 1999; Van Overwalle and Baetens, 2009). The morality dimension was selected to try to capture the association between certain words (e.g., “courage”) and the motivation to act in accordance with certain social or group rules, that has been hypothesized to reflect cognitive-emotional association complexes represented across a prefrontal cortex-temoro-limbic network (Moll et al., 2005). The executive function dimension was selected as certain words, particularly more abstract terms with multiple meanings or senses in different contexts, might be more frequently associated with activity in higher order cognitive systems mediating skills such as planning, selection, inhibition, executive flexibility, and strategizing (e.g., Stuss et al., 1995). The quantity dimension was selected as not only is the division between numerical and non-numerical semantics well-established, but also verbal terms which relate to quantity (e.g., quantifiers such as “many” and “few”) have been shown in individuals with semantic dementia to pattern more with numerical than linguistic concepts (Cappelletti et al., 2006). The time dimension was included because our subjective sense of time is fundamental to our psychology and conceptions of reality (Allman and Meck, 2012, p. 656) and the meaning of many words (e.g., “past,” “present,” “future,” “brief,” “lengthy”) are integrally linked to either temporal perspective or perception; however the relationship between such concepts and components of specific timing theories (e.g., scalar expectancy theory; Gibbon et al., 1984) remains unclear. The space dimension was assessed owing to previous work in aphasic stroke patients with refractory access disorders that has suggested that spatial information influences the organization of geographical concepts (Crutch and Warrington, 2003, 2010a); however, little is known about how spatial terms are mediated neurally (e.g., spatial metaphors) but it has been hypothesized that right posterior temporal and parietal cortices may be engaged in methaphoric extensions of spatial events (Chatterjee, 2008). Finally, the overall polarity of concepts (i.e., positive, neutral, negative) was also considered as a possible marker of the reward system (e.g., Rolls, 2000) because appraisal of stimulus valence is central to multiple goal-directed behaviors, and because valence may be linked to a range of stimulus attributes (e.g., spatial “up” and “down” information, as demonstrated in the space-valence congruence effect; Meier and Robinson, 2004). Naturally this is not an exhaustive list of cognitive dimensions which could have been assessed, and there is variability in the extent of the empirical and/or theoretical justification for including these particular dimensions in the current analysis. Dimension selection was also influenced by the practicalities of selecting dimension labels which were easily comprehensible and distinguishable for the lay participants providing the ratings.

At a more methodological level, collecting individual word ratings appears to offer a viable technique for examining the semantic attributes of abstract concepts. Certainly a number of techniques employed to study conceptual structure in the concrete domain are more difficult to translate into the abstract sphere. For example, feature listing, in which healthy individuals are requested to list physical and functional attributes of different entities, holds both intuitive and empirical appeal; hierarchical cluster analyses of the resulting data indicate the validity of the approach through the emergence of item clusters which correspond to recognizable taxonomic categories (e.g., fruit, vegetables, birds, etc.; Garrard et al., 2001; Cree and McRae, 2003; McRae et al., 2005; see Figure 1A). However, the feature listing approach is less easily applied to the domain of abstract words owing to the paucity of taxonomic terms, discrete properties, and other reliable verbal markers. For example, as the features of “cow” might include “is an animal,” “has udders” and “makes a mooing noise,” the equivalent features of abstract terms such as “victory” or “illusion” might be much more difficult to specify. Where abstract feature listing of abstract terms has been attempted, abstract terms have been claimed to have fewer intrinsic item properties, more properties expressing subjective experience, and properties which were less specific and more related to social aspects of situations [Wiemer-Hastings and Xu, 2005; see also Barsalou and Wiemer-Hastings (2005), for an exploratory attempt to investigate the content of three abstract concepts “truth,” “freedom,” and “invention”].

**Figure 1:**
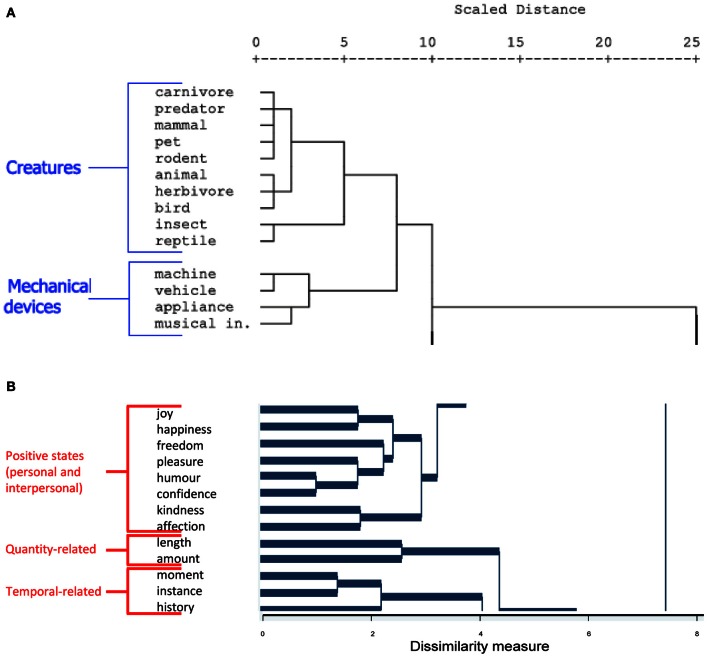
**(A)** Example labeling of dendrogram based on concrete item modality ratings [from Hoffman and Lambon Ralph (2012)]. **(B)** Labeling of dendrogram based on pilot 100-item abstract word ratings.

Instead of a feature generation method, the current study makes use of an abstract conceptual “feature” (ACF) rating which involves asking participants not to list features but rather to rate the importance of particular types of information to the meaning of a given word. Comparable Likert-scale-based rating approaches have been employed previously to explore the contribution of different sensory modalities to particular object categories (e.g., Gainotti et al., 2009; Hoffman and Lambon Ralph, 2012). However, to the best of our knowledge, this approach had not been applied to abstract words prior to our recent pilot studies (Crutch et al., 2012; Troche et al., 2012). Despite the similarity of these rating methods, differences in conceptual structure and availability of a taxonomic vocabulary between abstract and concrete concepts still make it difficult to directly equate studies of concrete and abstract features. This is illustrated by hierarchical cluster analysis of a pilot dataset of 100 abstract words rated for 9 of the cognitive dimensions listed above (Crutch, unpublished). The resulting dendrogram (see Figure 1B) reveals conceptual clusters that are intuitively coherent but less easy to label than the taxonomic clusters found in the concrete domain (see Figure 1A). Examples of words shown to cluster tightly together based on this ACF rating method include “vapor” and “illusion” which, introspectively, share an intangible quality, but nonetheless one which would be difficult to label or classify in a manner comparable to many concrete entities.

Using this ACF method, we have previously shown that some types of information are differentially important in the representation and organization of some types of abstract words (e.g., antonyms; Crutch et al., 2012). This study also demonstrated important differences between pairwise ratings of word similarity (often regarded as the gold standard for estimating semantic similarity in psycholinguistic research) and calculations of similarity based on individual word ratings. Pair-wise ratings (e.g., how similar are these two concepts) bias the rating toward a particular sense or meaning of the words involved, whereas individual ratings elicit data from which more flexible, context-independent semantic similarity metrics can be derived. For example, when completing ratings of antonyms (e.g., good-bad) and synonyms (e.g., good-great), participants' awareness that “opposites” should be maximally different clearly influenced their judgments on the pairwise similarity-ratings task (synonyms were given a much higher overall similarity rating than antonyms), whereas on the individual word ratings, antonyms were found to be as or even more similar than synonyms on every cognitive dimension except polarity.

The aim of the current study was to examine the utility of semantic similarity metrics derived from ACF ratings of abstract words. More specifically, a high dimensional semantic space was generated from control ratings of the contribution to individual abstract concepts of a number of different types of information: sensation, action, emotion, thought, social interaction, morality, time, space, quantity, and polarity. The validity of using inter-concept Euclidean distance within this high-dimensional space as a marker of semantic dissimilarity was then tested by evaluating whether these distances could predict the comprehension performance of a patient with global aphasia. We hypothesized that this patient would find it more difficult to discriminate between words located close together within the high-dimensional space than more distantly located concepts. The ACF Euclidean distance was also compared with Latent Semantic Analysis (LSA; Landauer and Dumais, 1997) cosine values representing word co-occurrence to determine which variable was the better predictor of patient performance. This examination of the semantic attributes of abstract words was motivated by the broader assumption that cognitive models of abstract conceptual knowledge must consider how both the relationships between abstract concepts and the attributes which constitute those individual concepts are represented.

## Case report

**Figure 2:**
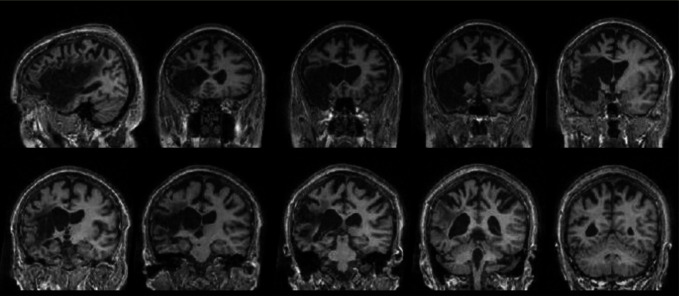
**MRI of SKO acquired 9 years post-stroke, demonstrating an extensive left fronto-temporo-parietal lesion.** Presented are a single sagittal slice, with nine coronal slices from anterior to posterior through the lesion area.

SKO is a 65-year-old male former chartered surveyor who developed global aphasia which resolved to a mixed non-fluent aphasia following a large left middle cerebral artery (MCA) territory stroke in 1997 (see Figure 2). The stroke resulted in an extensive left fronto-parietal infarct covering almost the entire MCA territory. Summary background neuropsychological information is provided in Table 1. SKO participated previously in a study of antonym comprehension (Crutch et al., 2012) and was selected for both studies on the basis of a linguistic profile that included deficits in verbal comprehension and impaired phonological–orthographic transcoding. SKO showed impaired performance on the British Picture Vocabulary Scale test of verbal comprehension, and in identifying the Crutch et al. (2007) high frequency items drawn from five categories. Furthermore, on a simple test of spoken non-word to written non-word matching, SKO scored near chance when the target and foil shared no phonemes or graphemes (e.g., “bep”-“bep” or “civ”: 7/10) and at chance when there was a single shared phoneme/grapheme (e.g., “bav”-“bem” or “bav”: 5/10). This transcoding deficit was necessary to enable the use of a simple spoken word to written word matching paradigm, involving the discrimination of two written words (e.g., “faith”-“faith” or “heresy”), as a measure of verbal semantic processing.

**Table 1:** **Summary neuropsychological information on patient SKO**.

		**SKO**
WASI matrix reasoning		18/32 (*T* = 49)
Digit span forwards		3 digits
Repetition		63/90
Reading [from Brown and Ure (1969)]		11/72
Graded non-word reading test		0/20
Spoken non-word–written non-word match		
Level I (e.g., bep-civ)		7/10
Level II (e.g., bem-bav)		5/10
British picture vocabulary scale (short)		26/32
Pyramid and palm trees test		
3 pictures		45/52
3 written words		34/52
McKenna and Warrington (1978)		
Naming	Animals	3/10 (30%)
	Man-made artifacts	3/10 (30%)
	Colors	2/10 (20%)
	Body part	3/10 (30%)
	Countries	9/10 (90%)
	TOTAL	20/50 (40%)
Comprehension	Animals	10/10 (100%)
	Man-made artifacts	10/10 (100%)
	Colors	7/10 (70%)
	Body part	6/10 (60%)
	Countries	10/10 (100%)
	TOTAL	43/50 (86%)

## Experiment 1—comparing the power of abstract cognitive feature and latent semantic analysis ratings to predict word comprehension performance

### Stimuli

The stimuli were drawn from a corpus of 400 nouns on which Abstract Cognitive Feature (ACF) ratings were previously acquired (Troche et al., unpublished). Of these 400 nouns half were classified as concrete and the other half as abstract based on imageability ratings (>500 or <450, respectively) from the MRC Psycholinguistic Database.

Following Crutch et al. (2012), participants were requested to rate individual concepts on 12 different dimensions using 7-point Likert scales. The Likert ratings from 7 (agree) to 1 (disagree) indicated participants' level of agreement with statements concerning the contribution to the concept in question of 9 different cognitive dimensions: sensation, action, thought, emotion, social interaction, morality, time, space, and quantity. Three further rating scales concerning the extent to which a concept was positive or negative (polarity) and the ease with which the concept could be modified^1^ or taught were also completed. A description of these parameters as presented to participants can be found in Appendix 1 (see also Troche et al., unpublished). Three hundred and sixty-five participants (Mean [SD]: Age = 40.8 [12.5]; Years of education = 15.3 [2.1]; 68% female) were recruited through the online program Mechanical Turk [see Buhrmester et al. (2011) for data on the validity and reliability of this approach] and rating surveys were created and completed within Survey Monkey (www.surveymonkey.com). Data were excluded if participants took less than 10 min to complete the survey, used less than half of the seven point Likert scale, or provided a run of more than 20 identical sequential responses.

For the current experiment, two independent symmetric matrices of pairwise semantic similarity ratings were derived for the 400 word set. Values in the first matrix denoted the Euclidean distance between words in a given pair based upon ACF ratings on the 12 dimensions specified above. The second matrix contained pairwise LSA (www.lsa.colorado.edu) cosines. A multidimensional scaling (MDS) map based on ACF ratings of the 400 words across all 12 dimensions is shown in Figure 3. A scatterplot showing the relationship between ACF distances and LSA cosines for all pairwise combinations of the rated abstract words (*N* = 208 words; 21,528 combinations) is shown in Figure 4. The two scales showed a modest correlation (*r* = −0.31), but a number of word pairs showed discrepant relatedness ratings [i.e., highly related on ACF but not LSA (e.g., metaphor-idiom) or vice versa (e.g., heresy-faith)].

**Figure 3:**
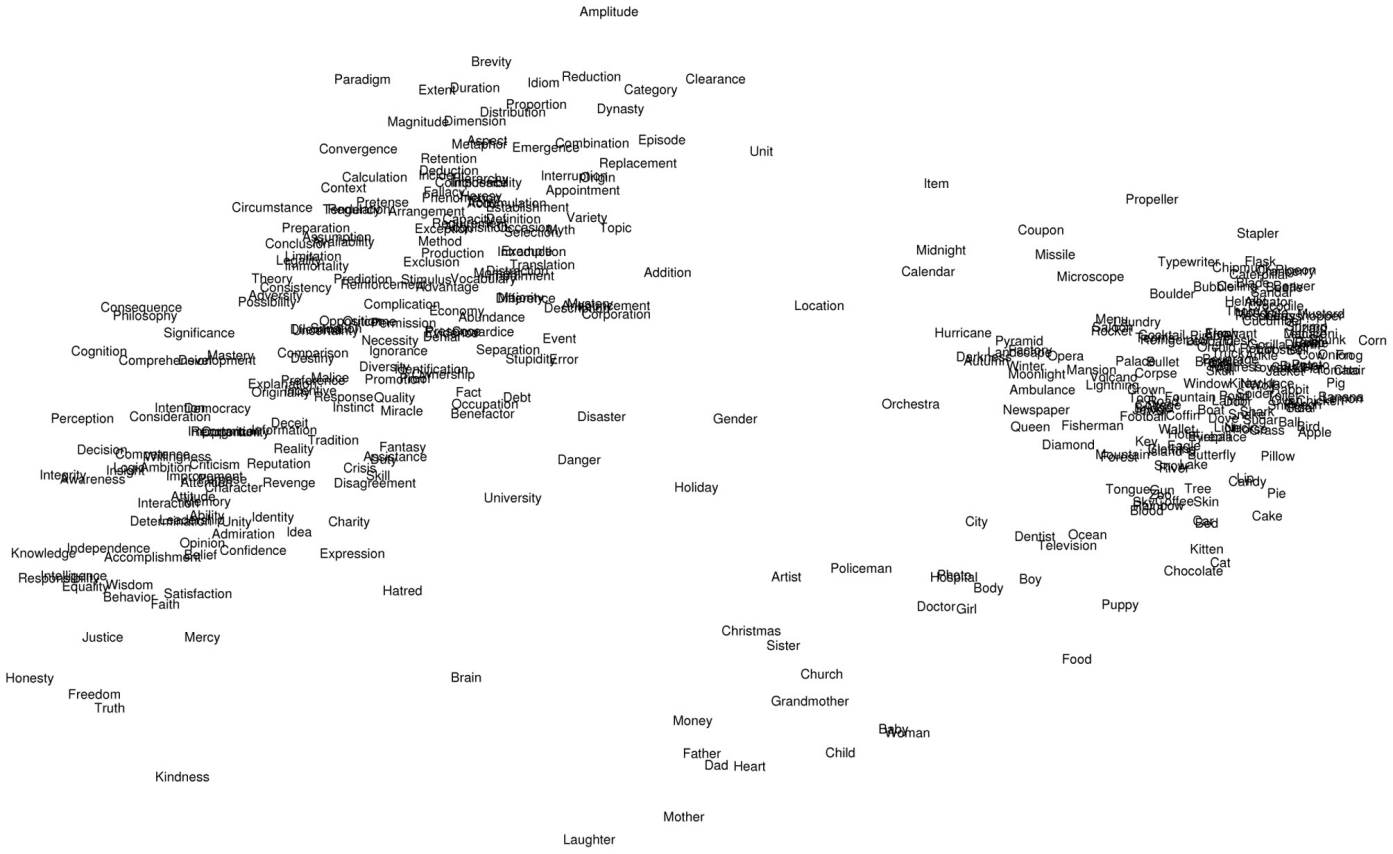
**Multidimensional scaling map based on ACF ratings of the 400 words across all 12 dimensions**.

**Figure 4:**
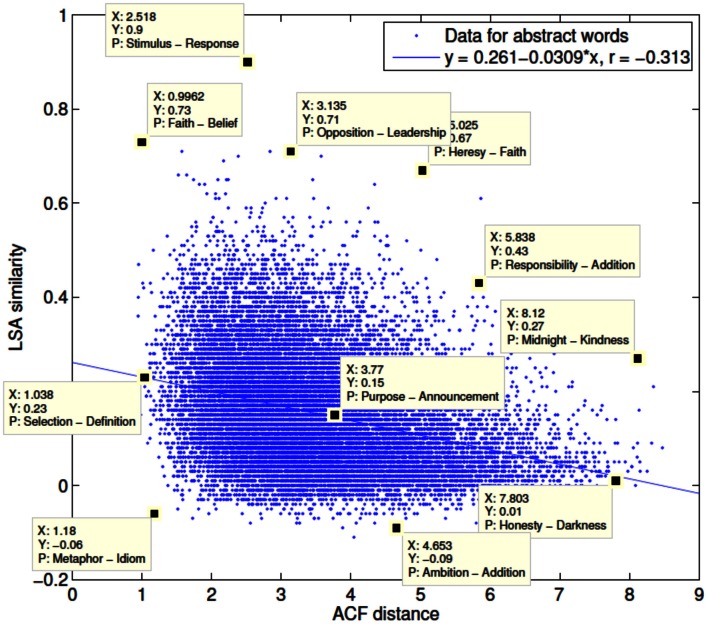
**Scatterplot showing the relationship between ACF Euclidean distances (lower values indicate greater relatedness) and LSA cosines (higher values indicate greater relatedness) for all pairwise combinations of the rated abstract words only (*N* = 208 words; 21,528 combinations)**.

For the purposes of stimulus selection, both ACF and LSA pairwise ratings underwent a linear transformation on to a common scale between 0 and 1 bounded by the minimum and maximum value in each matrix. The transformed LSA scale was also negated so that for each measure, low values indicate semantic relatedness (semantically close items) and high values indicate semantic un-relatedness (semantically distant items). The difference between the two matrices of transformed distances (ACF minus LSA) is referred to below as the ACF-LSA discrepancy matrix.

These matrices were then cut-down by excluding all concrete words (defined by a concreteness rating of more than 450 on the MRC Psycholinguistic Database; Coltheart, 1981). From these reduced matrices of abstract words, word pairs were selected under five conditions:

1. ACF maximum relatedness (ACFmax; *N* = 10)—most related words pairs from the ACF Euclidean values (irrespective of LSA ratings; e.g., attitude-belief).
2. LSA maximum relatedness (LSAmax; *N* = 10)—most related word pairs from the LSA ratings (irrespective of ACF ratings; e.g., opposition-leadership).
3. ACF more related than LSA (ACF > LSA; *N* = 10)—word pairs with highest values in the ACF-LSA discrepancy matrix (e.g., accumulation-majority).
4. LSA more related than ACF (LSA > ACF; *N* = 10)—word pairs with lowest (or most negative) values in the ACF-LSA discrepancy matrix (e.g., ignorance-truth).
5. Semantically unrelated (*N* = 10)—least related word pairs drawn equally from the ACF (ACFmin; *N* = 5) and LSA (LSAmin; *N* = 5) matrices (e.g., announcement-category).

In order to minimize overlap of individual words between conditions, the word pairs in each condition were selected from among the 20 highest/lowest rated pairs fitting each of the above descriptions. The mean raw, transformed and discrepancy ACF and LSA ratings are shown in Table 2. Furthermore, MDS plots of the distance between word pairs in each of the 5 experimental conditions are shown in Figure 5. As expected given the definition of the ACF > LSA and LSA > ACF conditions, there was no correlation between the ACF and LSA ratings for the 50 selected word pairs (*r* = −0.02). Additional data on the average concreteness, imageability, age of acquisition, frequency (Baayen et al., 1993) and length discrepancy of words in each pair are also given in Table 2. The concreteness and imageability of items differed between conditions [*F*_(4, 43)_ = 2.80, *P* = 0.04 and *F*_(4, 43)_ = 2.59, *P* = 0.05, respectively], but there were no overall significant differences between conditions of age of acquisition [*F*_(4, 33)_ = 1.72, *P* > 0.1], frequency [*F*_(4, 45)_ = 1.83, *P* > 0.1], familiarity [*F*_(4, 43)_ = 1.79, *P* > 0.1], or word length difference [*F*_(4, 45)_ = 0.10, *P* > 0.9].

**Table 2:** **Mean (and standard deviation) ratings for word pairs in each of the five conditions in Experiment 1; data are provided for ACF Euclidian distances, LSA cosines, adapted ACF and LSA ratings (where 0 is unrelated and 1 is related), ACF-LSA discrepancy (ACF adapted rating minus LSA adapted rating), concreteness (CNC), imageability (IMG), age of acquisition (A0A), frequency (CELEX), familiarity (FAM), and difference in number of letters (NLET)**.

**Condition**	**ACF euclidean**	**LSA cosine**	**ACF adapted**	**LSA adapted**	**ACF-LSA discrepancy**	**CNC**	**IMG**	**AOA**	**CELEX**	**FAM**	**NLET difference**
ACFmax	1.04 (0.06)	0.28 (0.16)	0.07 (0.01)	0.57 (0.15)	−0.50 (0.14)	280.2 (20.6)	326.2 (48.9)	501.4 (59.2)	41.1 (28.0)	517.6 (39.7)	2.3 (1.6)
ACF > LSA	1.72 (0.33)	−0.05 (0.06)	0.14 (0.03)	0.88 (0.06)	−0.74 (0.05)	314.0 (24.2)	337.7 (24.0)	512.3 (27.3)	14.1 (21.4)	473.0 (52.1)	2.3 (1.2)
LSAmax	2.88 (0.97)	0.70 (0.08)	0.26 (0.10)	0.19 (0.07)	0.07 (0.12)	324.9 (64.3)	402.6 (125.4)	457.9 (136.9)	40.4 (26.6)	512.9 (81.5)	2.0 (2.2)
LSA > ACF	5.20 (1.38)	0.45 (0.15)	0.49 (0.14)	0.42 (0.14)	0.07 (0.02)	306.7 (37.9)	351.1 (37.0)	459.6 (66.8)	28.0 (23.8)	530.6 (32.9)	2.5 (1.5)
Unrelated	5.60 (2.39)	−0.04 (0.11)	0.53 (0.24)	0.87 (0.10)	−0.34 (0.34)	339.7 (48.8)	405.3 (79.8)	402.9 (118.2)	31.9 (28.1)	528.3 (54.7)	2.4 (2.6)

**Figure 5:**
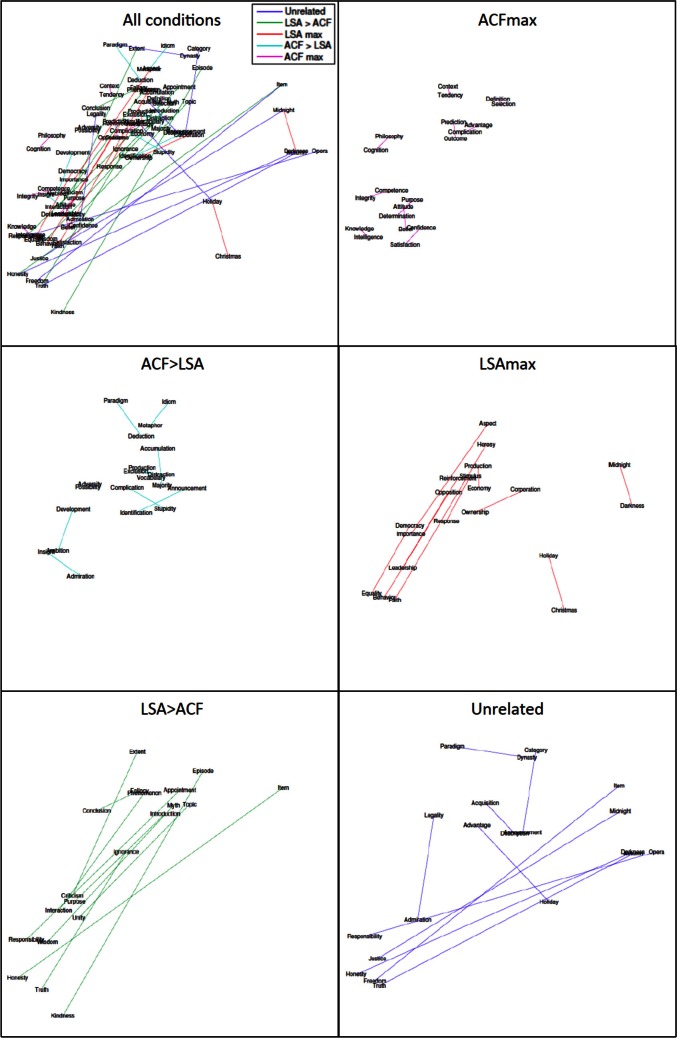
**Multidimensional scaling maps of the position of words constituting the word pairs tested in Experiment 1, showing plots for all conditions together and each condition separately**.

### Procedure

The identities of words in each pair were examined using a spoken word to written word matching paradigm. SKO was presented with a series of arrays comprising two written words. For each array the examiner spoke the name of one of these words aloud, with the identity of the target word varying between arrays in a fixed random order. On each occasion the patient was required to point to the word they had just heard. For each word pair, there were 10 consecutive trials, with each word probed five times in a pseudorandom order (maximum 3 consecutive presentations of the same target item). Written words were presented on opposite sides of the screen, with the position of words varied in a pseudorandom order so that target responses were on each side of the screen equally often (maximum 3 consecutive presentations of written words in the same spatial arrangement). Items were presented with an approximate 1 s response–stimulus interval (RSI) during which a blank screen was presented. This procedure yielded a total of 10 responses per word pair, and thus 100 in each condition, and 500 responses in total. Word pairs from each condition were presented in a pseudorandom order. The word pairs were presented on a MacBook Air laptop in the Print Preview mode of Microsoft Word in black 55 point Arial font on a white background.

### Analysis

Response accuracy was assessed using two complementary analyses owing to the lack of independence between responses inherent in the repetitive probing procedure. A logistic regression analysis of binary accuracy data for each response (*N* = 500) clustered by word pair was conducted with transformed ACF distance, transformed LSA cosine, concreteness, frequency and word length discrepancy as regressors. In addition, total scores were generated for each word pair (/10; *N* = 50) and analysed using linear regression with the same regressors. This latter model was also re-run replacing the ACF distance with the mean score differences for each of the 12 individual cognitive dimensions.

### Results

SKO's response accuracy in each of the five conditions is shown in Figure 6. Inspection of these raw data suggest that SKO responded less accurately in the ACFmax than LSAmax condition and less accurately in the ACF > LSA than LSA > ACF condition. Performance in the ACFmax and ACF > LSA conditions was worse than in the combined control condition but performance in the LSAmax and LSA > ACF conditions was comparable to the combined control condition. Dividing the combined control condition into the ACFmin and LSAmin sets, performance on the ACFmin stimuli was superior. Indeed performance on the ACFmin stimuli was superior to performance on all main experimental conditions (ACFmax, LSAmax, ACF > LSA, and LSA > ACF) whereas performance on the LSAmin stimuli was only superior to the ACFmax and ACF > LSA conditions.

**Figure 6:**
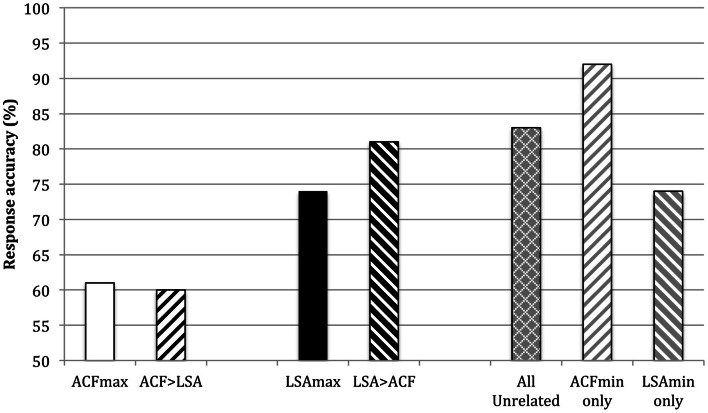
**Percentage correct responses in each of the following conditions: ACF maximum relatedness (ACFmax), ACF more related than LSA (ACF > LSA), LSA maximum relatedness (LSAmax), LSA more related than ACF (LSA > ACF), and semantically unrelated (data shown for both All unrelated, and separately for ACFmin and LSA min items)**.

The logistic regression of individual item response accuracy revealed a highly significant effect of semantic distance as defined by the ACF distance (*z* = 3.76, *P* < 0.001) but not LSA cosine (*z* = −0.03, *P* > 0.9). None of the control variable regressors had a significant effect upon response accuracy (concreteness: *z* = 0.67, *P* > 0.5; frequency: *z* = 1.99, *P* > 0.05; difference in word length: *z* = −0.88, *P* > 0.3).

The results of the linear regression analysis of word pair scores revealed similar results, with a significant effect of semantic relatedness as defined by ACF distance (*P* = 0.005) but none of the other regressors (LSA: *P* > 0.7; concreteness: *P* > 0.5; frequency: *P* > 0.1; difference in word length: *P* > 0.4). When this model was repeated using the mean discrepancy scores for each of the 12 individual cognitive dimensions instead of the ACF distance, none of the individual rating discrepancies were found to be a significant predictor of SKO's response accuracy.

We also examined whether these apparent differences between comprehension accuracy for words selected on the basis of the ACF and LSA ratings were evident on the first response to each target. The number of correct responses was calculated for ACF (summing across ACFmax and ACF > LSA), LSA (summing across LSAmax and LSA > ACF), and unrelated items (summing across ACFmin and LSAmin). Chi-squared tests revealed performance was significantly worse in the ACF condition than the unrelated condition (χ^2^_[1]_ = 4.26, *P* = 0.04), but neither of the remaining comparisons was significant (LSA vs. unrelated: χ^2^_[1]_ = 1.88, *P* > 0.1; ACF vs. LSA: χ^2^_[1]_ = 0.95, *P* > 0.3).

### Comment

The ACF distance metric, based on control ratings of the contribution of different cognitive dimensions to each concept, was the only significant predictor of SKO's response accuracy. This suggests that these novel ratings captured important aspects of the conceptual relationship between the two words in each pair which were not captured as strongly by the co-occurrence-based LSA cosine. It is of note that none of the 12 individual rating differences were found to be a significant predictor of performance; only distance within the high-dimensional space generated from these ratings predicted response accuracy.

## Experiment 2—non-repetitive probe comprehension task

The data reported in Experiment 1 indicate that the ACF distance is a predictor of SKO's ability to discriminate two words. In this Experiment, we tested the complementary null hypothesis, that words matched closely for distance would yield comparable levels of patient response accuracy. In particular, we tested whether this held true even when the words being examined were drawn from different areas of the semantic space as defined by representation at different ends (high/low) of an individual rating scale. In this case the “quantity” rating scale was selected as this was the single dimension which approximated most closely to one of the three factors (perceptual salience, emotion/social cognition, and magnitude) which emerged from the hierarchical cluster analysis of all 400 words in the original corpus (Troche et al., unpublished). However, equivalent results would be predicted had another dimension been selected as a means of defining different regions within the semantic space.

### Stimuli

All abstract words (CNC rating <450) from the Troche et al. set were rank ordered by their ratings on a single dimension: quantity. The 20 words with the highest quantity ratings and the 20 words with the lowest quantity ratings were selected. From these, two sets of 16 words were selected, and within each set words were formed into word pairs. Critically the mean ACF distance between words in high and low quantity word pairs was matched (i.e., they were very closely matched for the ACF rating of semantic relatedness; *t* = 0.004, *P* > 0.99, 2-tailed test). High and low quantity words were also matched for concreteness, imageability, age of acquisition, frequency, familiarity and number of letters, phonemes, and syllables (all *P* > 0.05, 2-tailed test; see Table 3).

**Table 3:** **Mean (and standard deviation) ratings for high and low quantity items (Experiment 2) on multidimensional ACF semantic ratings (ACFdist), concreteness (CNC), imageability (IMG), age of acquisition (A0A), frequency (CELEX), familiarity (FAM), and number of letters (NLET), phonemes (PHN) and syllables (NSYL)**.

	**ACFdist**	**CNC**	**IMG**	**AOA**	**CELEX**	**FAM**	**NLET**	**NPHN**	**NSYL**
High quantity items	1.90 (0.26)	311.2 (52.0)	359.2 (58.7)	485.9 (42.3)	32.0 (30.4)	514.6 (42.4)	8.6 (1.9)	7.9 (1.7)	3.1 (0.8)
Low quantity items	1.88 (0.44)	317.5 (54.4)	377.1 (98.7)	482.4 (106.5)	14.3 (20.4)	477.7 (76.5)	7.7 (3.0)	6.6 (2.8)	2.8 (1.1)

### Procedure

The task involved spoken word to written word matching as in Experiment 1, except that each item was only probed once per block. Within each block, all written word pairs were presented twice in a pseudorandom order, once with the spoken name of one written word and once with the spoken name of the alternate word (*N* = 8 word pairs and *N* = 16 spoken word targets per block). All low quantity items were presented in the first block, and all high quantity items presented in the second block. Later in the testing session, both blocks were repeated in the reverse order with a different within-block pseudorandomized trial order. This yielded a total of 32 responses in each condition.

### Results and comment

SKO showed identical response accuracy rates for the two conditions (High quantity words = 21/32, Low quantity words = 21/32). This result supports the conclusion drawn from Experiment 1 that distance within the ACF high-dimensional space can provide reasonable metric of semantic relatedness, at least in relation to the comprehension performance of patient SKO. The close matching of accuracy levels across words drawn from different areas within that semantic space also suggest that this metric may have utility for determining/predicting semantic relatedness among a diverse set of concepts.

### Discussion

The aim of this study was to evaluate a novel metric for measuring the semantic relatedness of abstract words. The study relates to the current Frontiers Research Topic on sensorimotor processing and (abstract) conceptual knowledge because the abstract cognitive “feature” (ACF) ratings described are based on control estimates of the contribution of different cognitive systems to individual concepts. These cognitive dimensions include those central to strong embodiment theories of cognition (sensation and action), additional domains posited by weak embodiment theorists (emotion), and other types of information not previously considered in this regard (labeled: thought, social interaction, morality, time, space, quantity, and polarity), plus ease of modifiability and teaching. These ratings were designed to measure the content or semantic attributes of abstract words, and thus to be loosely analogous to feature generation approaches to the study of the structure of concrete conceptual knowledge (e.g., Garrard et al., 2001; Cree and McRae, 2003). However, like recent attempts to rate the contribution of different modalities to concrete concepts (Gainotti et al., 2009; Hoffman and Lambon Ralph, 2012), the ACF approach avoids the constraints of linguistic labels inherent in feature generation. The approach also benefits from the consideration of concepts individually rather than generating (context dependent and less flexible) pairwise ratings of the specific relationship between two words. The ACF approach described was also intended to complement rather than compete with measures of word co-occurrence such as LSA that better capture linguistic experience and contextual association.

In Experiment 1, we hypothesized that if the high-dimensional space generated from ACF control ratings approximates the organization of abstract conceptual space, then words separated by small Euclidean distances should be more semantically related than words separated by greater distances, and should therefore be more difficult to distinguish for our patient with a comprehension deficit, SKO. It should be emphasized that ACF semantic space is based on numerical ratings for individual words not word pairs, and therefore none of judgments gathered from controls correspond directly to the relationship between the word pairs used in Experiment 1. As predicted, SKO was significantly worse at identifying targets presented within word pairs with low ACF distances. Neither LSA cosines nor any of the background variables were found to be significant predictors of response accuracy. SKO's performance on this spoken word to written word matching task is indicative of semantic processing as his phonological to orthographic transcoding route is so impaired he is forced to make responses on the basis of words' semantic properties (e.g., even in the unrelated condition, SKO occasionally made errors distinguishing items with highly distinct phonological and orthographic forms, such as “opera—responsibility”).

In Experiment 2, we tested the complementary hypothesis that word pairs matched closely for ACF distance would yield equivalent levels of response accuracy. The critical aspect of this otherwise rather drab-sounding experiment was to select items from different areas within the high-dimensional ACF space, namely words rated at opposite ends of a particular rating dimension, quantity. Again as predicted, SKO's response accuracy was perfectly matched across the two conditions. This suggests that the distance provides some measure of word relatedness across quite a diverse array of topics and subject areas. The failure of word pairs constructed from two words both rated highly for a single variable to yield a higher error rate in SKO is also consistent with the idea that it is the combination of different cognitive dimensions rather than any single dimension that contributes to the predictive power of the ACF distance in the current mixed set of abstract words. More direct support for this may be taken from the failure of any single rating dimension to predict SKO's response accuracy in Experiment 1.

Taken together with previous evidence of the explanatory power of ACF ratings for antonym and synonym discrimination in aphasic patients (Crutch et al., 2012), these experiments provide preliminary support for an approach which attempts to quantify the semantic similarity of abstract words based on their constituent semantic attributes rather than their specific, contextually-bound relationships to other abstract words. It should be noted that the data presented does not distinguish between embodied and disembodied theories of conceptual knowledge. The broader approach for which the data argues, namely that a number of different types of information and internal experience contribute to abstract conceptual knowledge, could be incorporated within all but strong embodiment positions. However, given the published literature on the topic of embodiment and our clinical experience working with semantic dementia patients, our working assumption is that conceptual knowledge does require some form of abstract representation (in line with the disembodied and grounding by interaction positions). The corresponding working hypothesis is that the types of information discussed in the current study (e.g., emotion, social interaction, quantity, polarity) influence the acquisition and organization more than the retrieval of abstract conceptual knowledge.

Several caveats and questions regarding the ACF methodology should be raised. First, the cognitive dimensions outlined here (e.g., quantity, polarity) are not equivalent to the sensory modalities referenced in strong embodiment theories (e.g., vision, audition), in that they reflect secondary or higher-order associative processing of information acquired through the primary sensory modalities. The activity of these brain networks may not constitute “embodiment” in the literal sense described for the sensorimotor networks, but two points are of relevance here. The embodiment/disembodiment debate is not binary in nature (Meteyard et al., 2012); “weak” embodiment positions have been advanced which highlight the contribution of emotion/affect (Kousta et al., 2011), another form of higher order information whose acquisition and/or activation in response to internal and external stimuli is often mediated by primary sensory systems. In addition, non-embodiment theorists argue that much of the evidence cited in favor of embodied cognition in fact reflects interactions not with primary sensory cortices but higher-order polymodal cortices (Bedny and Caramazza, 2011). In the light of these two lines of (opposing) argument, the weak embodiment position could potentially incorporate other types of information outside of the primary senses (e.g., magnitude). Alternatively, under a more disembodied framework, these additional cognitive dimensions could be regarded as influencing the organization of conceptual knowledge during acquisition and interacting with conceptual representations when activated.

Second, as noted above, the ACF ratings are for individual words rather than word pairs, yielding the advantages of context-independence and greater flexibility. However, many words have different meanings (homonymy) and/or senses (polysemy), and no precise definition was provided to control participants. Consequently participants may have had slightly different meanings in mind when rating each item. As a result the position of each word within the high-dimensional space should be regarded as an estimate of the “true” locus of each homonymous/polysemous word, and the distance between pairs of words may have greater validity for some meanings than others.

Third, concrete semantic space remains rather under-elaborated owing to (deliberate) selection of dimensions likely to pertain to abstract concepts (see Figure 3). Previous feature generation studies have highlighted a number of different types of knowledge more germane to the concrete domain (e.g., visual—color, visual—parts and surface properties, visual—motion, tactile, olfactory, gustatory, auditory, functional, and encyclopedic; Cree and McRae, 2003). These dimensions could easily supercede the broad “sensation” and “action” dimensions used in the current ratings. This approach might yield a more comprehensive set of “feature” information about concrete concepts, the richness of which would be more suitable for comparison of concepts across the entire concreteness spectrum.

Fourth, differences likely exist between the dimensions rated. For example, the dimension labels used were deliberately non-technical lay terms (e.g., social interaction) so the directness of the mapping between the labeled dimension and the type of information to which it was intended to refer may vary between dimensions. Naturally the list of dimensions employed in the study was also not exhaustive with, for example, no explicit reference to episodic memory. It has also been suggested recently that abstract concepts may also depend in part upon brain circuits involved in introspection (Van Overwalle and Baetens, 2009; see Kiefer and Pulvermüller, 2012), which may relate to one or more the rating dimensions used in the current study.

Fifth, the ACF rating approach was developed to examine the notion that domains of cognition beyond the realm of sensorimotor and emotional processing may play an important role in the acquisition and/or organization of conceptual knowledge. However, the current study represents only one stage in the examination of this broad hypothesis, namely evaluating whether the ratings yield a viable metric of semantic distance between abstract word concepts. The data do not, and were not intended to, provide any direct (neural) evidence that the pattern of comprehension performance observed in SKO is linked causally or non-causally to the activation of these cognitive systems.

One final point worth clarifying is that we regard the “feature”-based similarity data presented in the current paper to be complementary to rather contradictory of previous claims about the relatively greater importance of association than similarity for abstract words (e.g., Crutch and Warrington, 2005, 2010b; Crutch and Jackson, 2011). From the outset, the theory of qualitatively different representational frameworks was proposed to describe a relative rather than absolute distinction between the qualitative representational structures supporting abstract and concrete words. As stated in the Introduction, to understand abstract conceptual knowledge we need not only to investigate the relationships between abstract concepts but also to explore of what those different individual concepts are composed. To that end, the current study builds on a small number of previous attempts to directly compare the features of abstract and concrete words (e.g., Wiemer-Hastings and Xu, 2005; Connell and Lynott, 2012). After all, whilst the meaning of abstract words may be shaped by the context in which they occur, they may also be understood in isolation or in unfamiliar or incompatible contexts.

### Conflict of interest statement

The authors declare that the research was conducted in the absence of any commercial or financial relationships that could be construed as a potential conflict of interest.

## Footnotes

^1^One influential approach to abstract word representation has emphasized the role of context availability in facilitating lexical access for concrete words (Schwanenflugel and Shoben, 1983; Schwanenflugel et al., 1992). Concrete words are thought to more readily stimulate a linguistic context relative to abstract words, especially for words appearing in isolation (Schwanenflugel and Shoben, 1985). One variable related to context availability is the ease with which a word can be modified or predicated. For example, a concrete word such as giant is easily modified (e.g., big, friendly, scary giant) whereas abstract words (e.g., fate) often lack such a structure. We measured ease of modification as a way of quantifying this lexical characteristic.

## Appendix

**Appendix 1:** **The wording and anchor points for the 7-point Likert Scales used to rate the target words on each of the 12 dimensions**.

**Parameter**	**Definition**
Polarity	I relate this word to positive or negative feelings in myself.
Sensation	I relate this word to physical feelings like vision, hearing, smelling, etc.
Action	I relate this word to actions, doing, performing, and influencing.
Thought	I relate this word to mental activity, ideas, opinions, and judgments.
Emotion	I relate this word with human emotion.
Social interaction	I relate this word with relationships between people.
Time	I relate this word with time, order, or duration.
Space	I relate this word to position, place, or direction.
Quantity	I relate this word to size, amount, or scope.
Morality	I relate this word to morality, rules or anything that governs my behavior.
Ease of modifying	I can easily choose an adjective for this word (the ugly truth, whole truth, etc.).
Ease of teaching/learning	This word could be easily taught to a person who does not speak English.
